# Dynamic-locking-screw (DLS)–leads to less secondary screw perforations in proximal humerus fractures

**DOI:** 10.1186/1471-2474-15-194

**Published:** 2014-06-04

**Authors:** Thomas Freude, Steffen Schroeter, Michael Plecko, Christian Bahrs, Frank Martetschlaeger, Tobias M Kraus, Ulrich Stoeckle, Stefan Doebele

**Affiliations:** 1 Department of Traumatology and Reconstructive Surgery, BG Unfallklinik, Eberhard Karls Universitaet Tuebingen, Schnarrenbergstrasse 95, 72076 Tuebingen, Germany; 2 Department of Traumatology, University Hospital Zurich, University of Zurich, Raemistrasse 100, CH-8091 Zurich, Switzerland; 3 Department of Orthopeadic Sports Medicine, University Hospital rechts der Isar, Technische Universitaet Munich, Munich, Germany

**Keywords:** Dynamic-locking-screw 3.7, Proximal humerus fracture, Locking compression plate, Fracture healing

## Abstract

**Background:**

Loss of reduction and screw perforation causes high failure rates in the treatment of proximal humerus fractures**.** The purpose of the present study was to evaluate the early postoperative complications using modern Dynamic Locking Screws (DLS 3.7) for plating of proximal humerus fractures.

**Methods:**

Between 03/2009 and 12/2010, 64 patients with acute proximal humerus fractures were treated by angular stable plate fixation using DLSs in a limited multi-centre study. Follow-up examinations were performed three, six, twelve and twenty-four weeks postoperatively and any complications were carefully collected.

**Results:**

56 of 64 patients were examined at the six-month follow-up. Complications were observed in 12 patients (22%). In five cases (9%), a perforation of the DLS 3.7 occurred.

**Conclusions:**

Despite the use of modern DLS 3.7, the early complications after plating of proximal humerus fractures remain high. The potential advantage of the DLS 3.7 regarding secondary screw perforation has to be confirmed by future randomized controlled trials.

## Background

Proximal humerus fractures in elderly patients account for approximately 5% of all fractures and are mainly related to osteoporosis [1,2]. The treatment of displaced and unstable 3-/4-part fractures remains controversial. Techniques for osteosynthesis of proximal humerus fractures include closed or open reduction, and fixation with pins, plates, or intramedullary nails in previous studies, the clinical results have not shown to be very predictable [3-8]. Krappinger et al. identified local low bone-mineral-density (BMD), high age and anatomic reduction without medial cortical support as the main factors leading to unfavourable outcomes [9].

In order to improve the anchorage of screws especially in osteoporotic bone, angular stable proximal humerus plates have been invented in recent years. Many authors agreed these implants to be an important advance in the treatment of osteoporotic proximal humerus fractures [10-13]. However, screw perforation became a typical problem. According to the literature, screw perforation rates range from 4.9% to 23% [11,14,15].

Recently, the Dynamic-Locking-Screw 3.7 (DLS 3.7,DePuy-Synthes, Inc. 1302 Wrights Lane East West Chester, PA 19380. USA) was developed to decrease the high rigidity of standard locked plate constructs. The new design of the DLS 3.7 offers the following advantages:

1. Compared to the Locking-Head-Screw 3.5(LHS 3.5), the DLS 3.7 has a blunt tip.

2. The core diameter of the DLS 3.7 has increased.

3. Based on the biomechanical data of our pre-study, we assume that the pin-sleeve design leads to a more homogenous stress distribution over the length of the screw.

4. By reducing the difference between bone and implant stiffness it potentially reduces the force peak on single screws and enables a more homogenous force distribution.

The purpose of the present multi-centre study was to evaluating the early clinical results and complications after the treatment of proximal humerus fractures using a PHILOS plate (DePuy-Synthes, Inc. 1302 Wrights Lane East West Chester, PA 19380. USA) in combination with the modern DLS 3.7.

## Methods

Between 03/2009 and 12/2010, a multi-centre study in 5 level I trauma centres on plating of proximal humerus fractures with the PHILOS plate and the DLS 3.7 was conducted. The study population consisted initially of 64 patients suffering from a fracture of the proximal humerus. After the minimum follow-up of 6 months 56 patients remained, 8 patients didn’t come for the final follow up exams and were secondary excluded. Surgery was performed at one of the included level I trauma centers. Exclusion criteria were re-fracture, chronic inflammatory diseases, cancer, medication of immune response modulating drugs, HIV or chronic hepatitis B/C, allergic reaction to the Cobalt-Chromium-Molybdenum alloy (CoCrMo). All participating patients signed an informed consent. Clinical and radiological follow-up exams were performed at 3, 6, 12 and 24 weeks. The fracture of each patient was classified according to the AO Classification and Neer Classification of fractures. The length of the plate, the type and the number of screws were documented. The quality of reduction was validated post operatively by x-ray in axial and true- a.p. view. Furthermore, the fracture healing, the implant-specific complications (screw loosening, breakage and screw perforation), the general complications and the pain perception according to the VAS (Visual-Analogue-Scale) were recorded.

### Dynamic Locking Screw (DLS)

**Figure 1:**
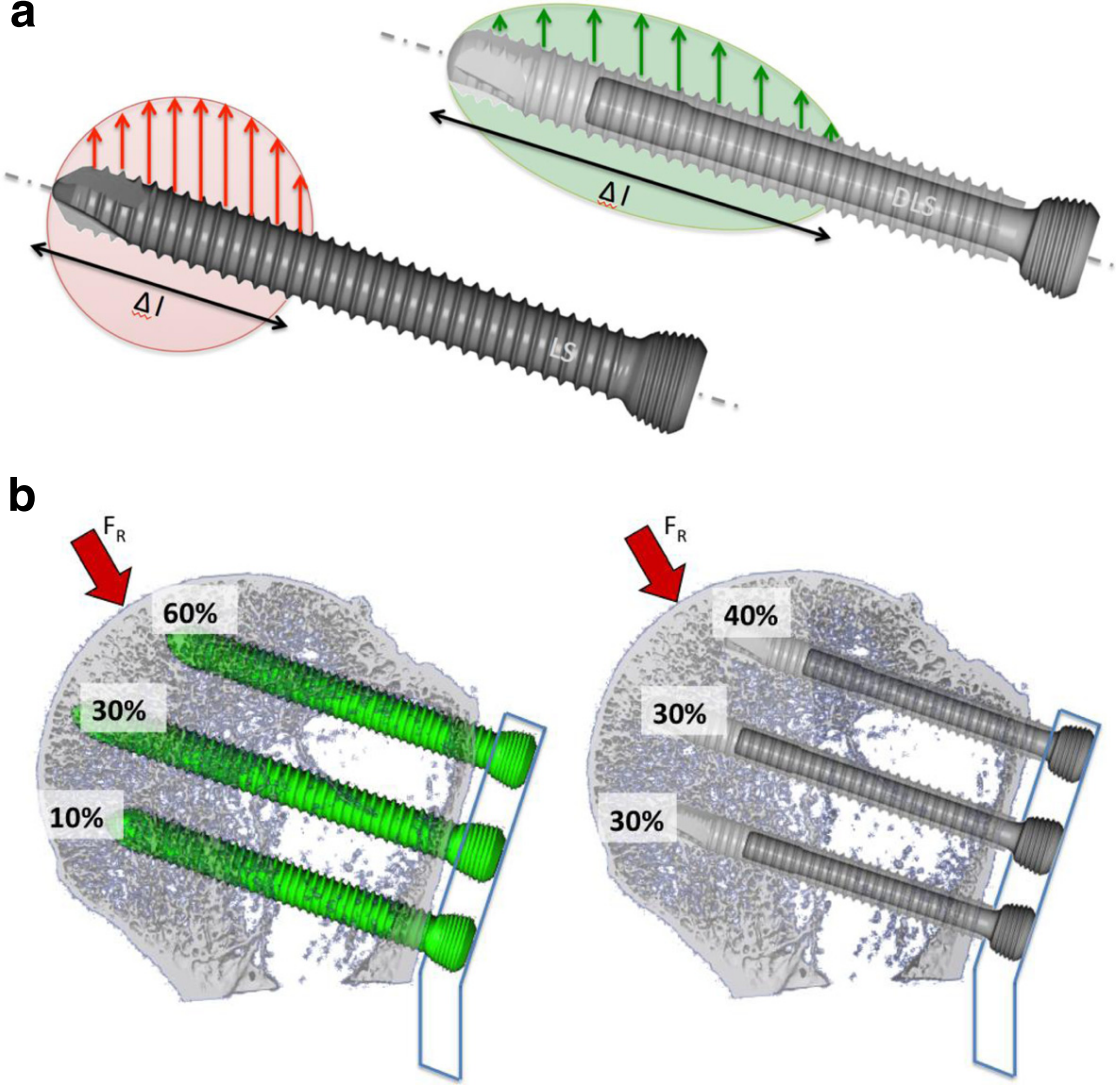
**Screw lead distribution (DLS 3.7 vs. LHS 3.5) schematic drawing. a**: Intra-screw; **b**: Inter-screw.

The DLSs are a new generation of locking screws that enables the surgeon to control the rigidity of plating constructs. It builds on the proven advantages of standard locking screws by eliminating tension on the bone and the compression between the plate and the bone, while simultaneously retaining the blood circulation and protecting the periosteum from potential damage. The DLS 3.7 consists of a pin with a threaded screw head, which is melted into a threaded sleeve (Figure 1) based on the angular-stable technique of Locking-Head-Screws. The DLS 3.7 is made of Cobalt-Chromium-Molybdenum alloy (CoCrMo). The material of the screw was changed in order to increase the strength under cyclic forces, especially near the head of the screw and in the welding area between the pin und the sleeve, and in order to reduce the bone-screw interface for better implant removal. This screw material can be combined with TAN or stainless steel plates without any biocompatibility problems.

The handling of this drill sleeve and its drillbit remained unchanged in comparison to the standard tools. The DLS 3.7 can be either inserted with a power tool or manually, but always in combination with a Torque Limiter 1.5 Nm. When problems arise with the insertion of the DLS 3.7, the use of the procedure that was already established for the LHS 3.5, was recommended. For the LHS 3.5, the standard instruments in combination with the 3.5 LCP were used.

The increased core diameter of the DLS 3.7 with the blunt tip design fits all 3.5 LC-Plates. Biomechanical tests could clearly demonstrate the effect of reducing the rigidity of the screw plate interface [16]. This is a particularly beneficial feature when bridging chosen as a method for the fracture treatment.

### Surgical procedure

Each surgery was performed within 4 days after the trauma. The PHILOS plate, which is available at different lengths, was inserted at the site of fracture. In the study protocol, no limitation was fixed for the number of the used LHS or DLS 3.7. In the framework of this study, it was intended to use the DLS 3.7 at least in the head-part of the PHILOS-plate, or in the head and the shaft. A mixture of screws (DLS 3.7 and LHS 3.5) on one fracture side, might it be in the humerus head or the shaft was not allowed. All inserted screws in the humerus head (position A to E in the plate) the screw tip was placed at a distance of at least 2 mm from the subchondral zone. The standard approaches to the proximal humerus were used, either the deltoid-pectoral or the delta-split approach [12].

## Results

The mean age of the patients was 60 ± 16 years (39% male, 61% female). According to the AO-Classification and the Neer-Classification, we included the fracture types listed in Tables 1 and 2.

**Table 1:** AO Classification in proximal humerus fracture

A	21
B	17
C	18

**Table 2:** Neer classification

		**2-part**	**3-part**	**4-part**
**Neer I**				3
**Neer II**	anatomical neck	5		
**Neer III**	surcical neck	16		
**Neer IV**	greater tuberosity		10	5
**Neer V**	lesser tuberosity		5	7
**Neer VI**	fracture dislocation		2	3
**Total**		21	17	18

Additional Fibre Wire fixation of the rotator cuff was used in 55% of the patients, while a tenotomy of the long biceps tendon was performed in 75% of the cases. In total, 435 DLS 3.7 were used. On average, 7.2 ± 1.6 screws per patient were implanted in the proximal part, while 2.6 ± 1.0 were used in the distal part. Furthermore, 1.3 ± 0.9 LHS 3.5 were used per patient and the mean number of small fragment cortical-screws was 1.2 ± 1.1 per patient.

**Figure 2:**
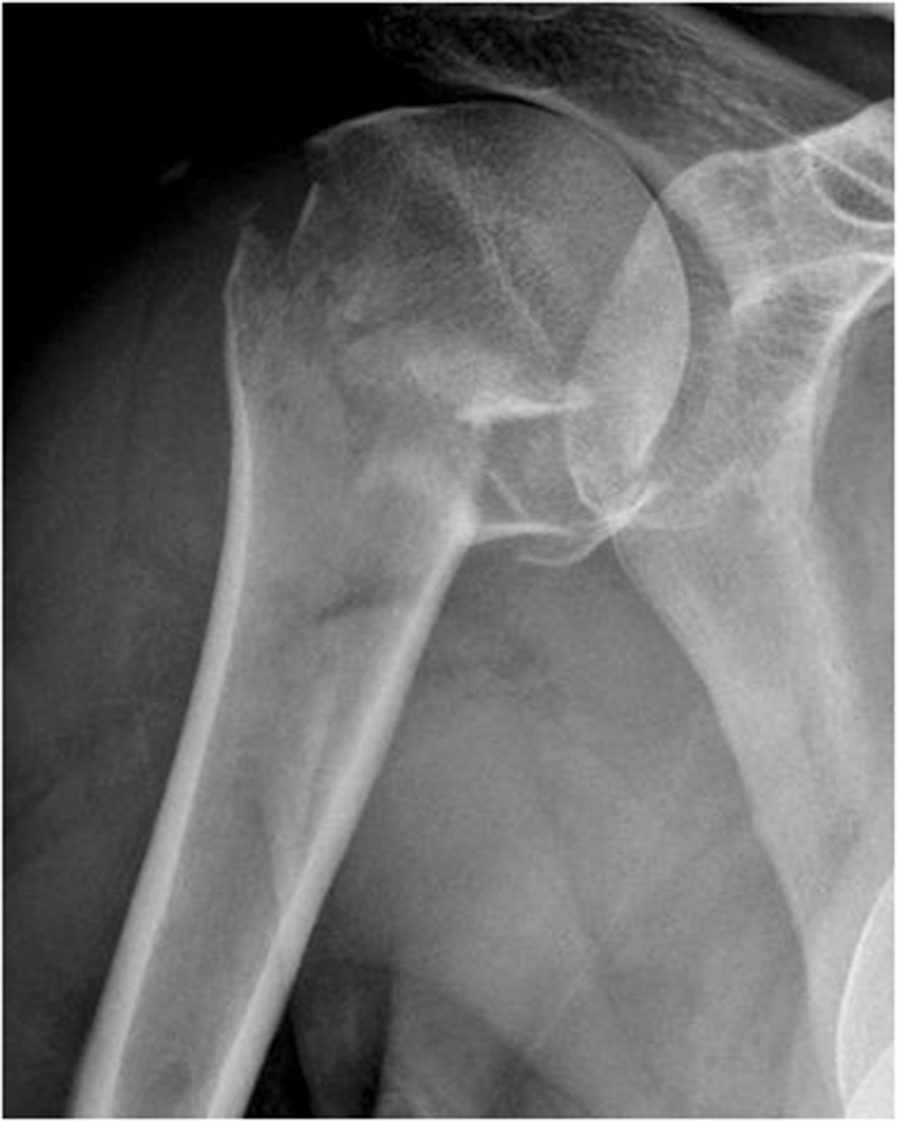
Proximal humerus fracture: preoperative.

**Figure 3:**
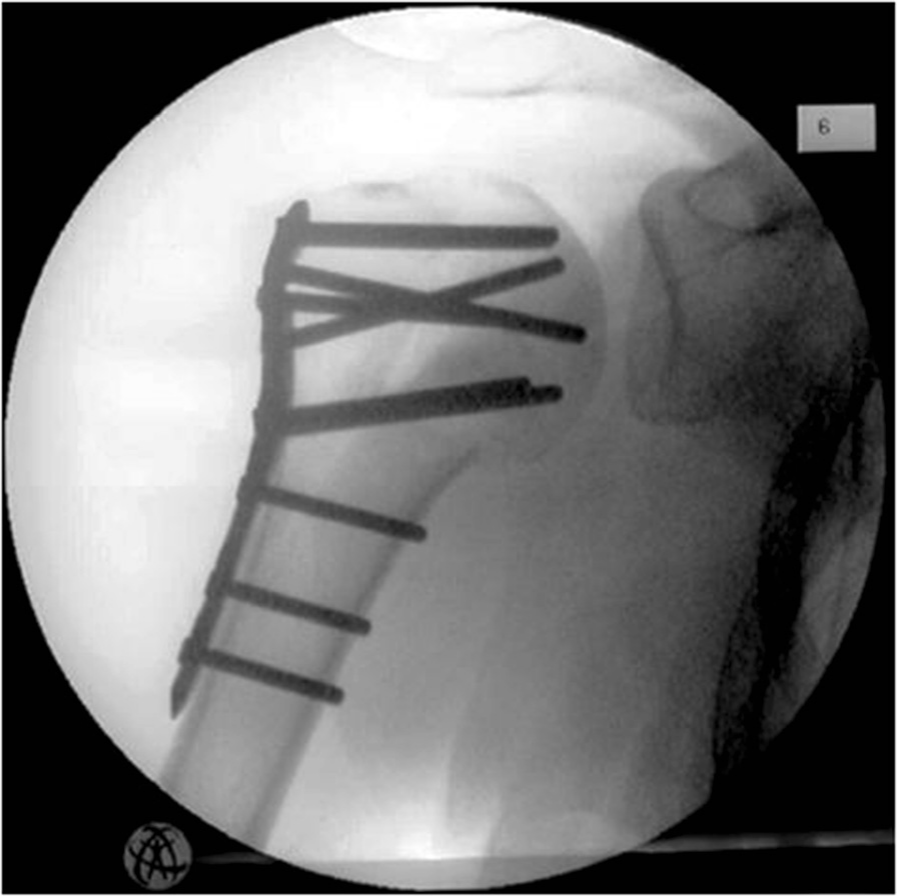
Proximal humerus fracture: postoperative.

**Figure 4:**
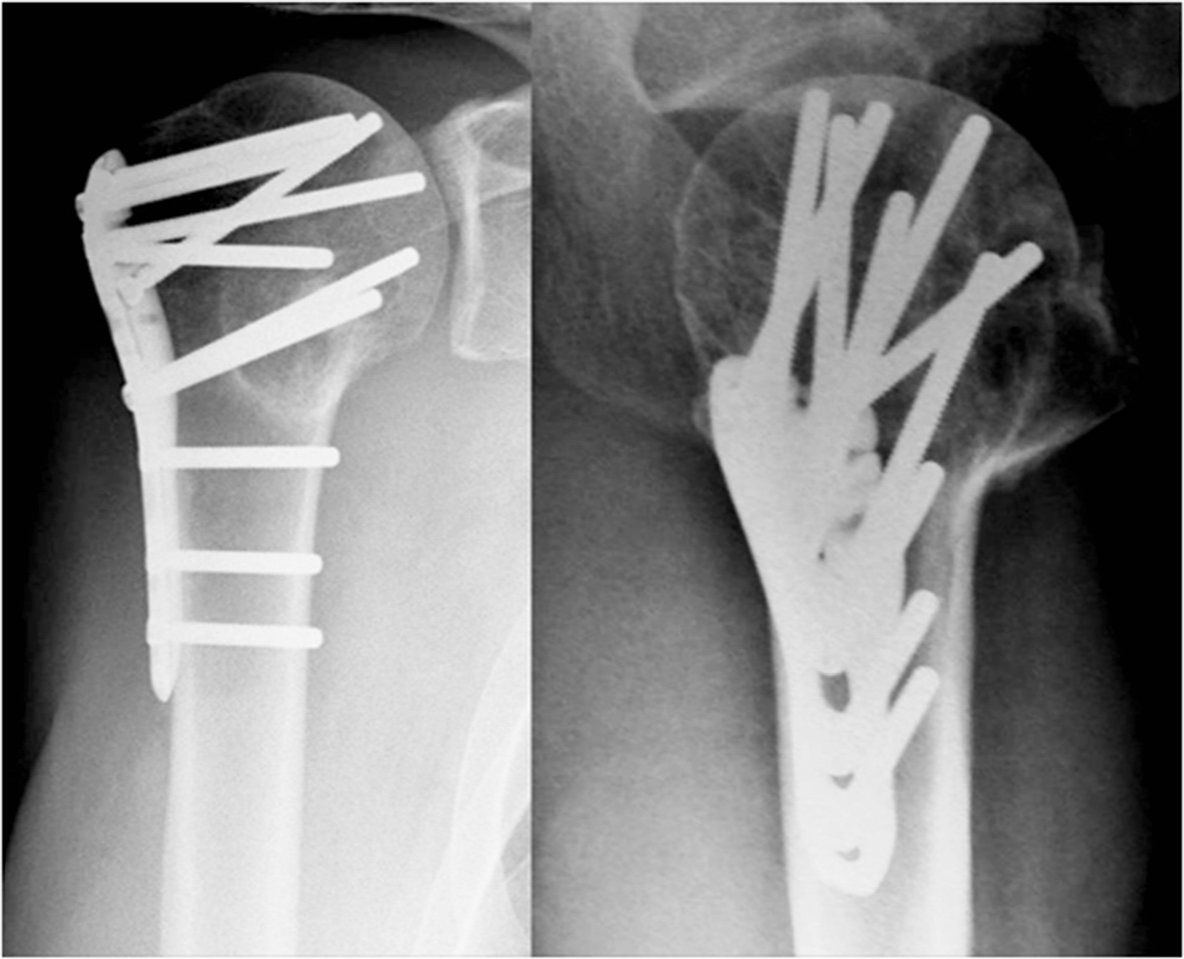
Proximal humerus fracture at the 24 weeks follow-up.

The fracture gap was observed in 86% of the patients after 3 weeks, in 71% after 6 weeks, in 27% after 12 weeks, and in 8% after 24 weeks. 3 weeks after the surgery, the mean VAS was 3.5 ± 1.5 and improved to 2.7 after 6 weeks (p < 0.0179). After 3 months, the mean VAS was 2.5 and improved to 2.3 after 6 months (n.s.). This improvement depended on the fracture type. However, after 6 months, the VAS was almost the same in all types of fracture (Table 3).Figures 2, 3 and 4 show the follow up of one patient treated with DLS 3.7.

**Table 3:** VAS according AO Classification in proximal humerus fracture

**AO Müller classification of fracture**	**3 weeks**	**6 weeks**	**3 months**	**6 months**
**A**	2.6 ± 0.5	1.9 ± 0.9	2.4 ± 1.6	2.4 ± 1.6
**B**	3.8 ± 2.8	2.6 ± 1.3	2.5 ± 1.3	1.8 ± 1.1
**C**	4.0 ± 1.0	4.2 ± 1.9	3.2 ± 2.3	2.7 ± 2.0

### Complications

Complications were observed in 12 (22%) patients. In three cases (6%), a dislocation of a fragment was observed. Three secondary displacements, two of the greater tuberosity and one varus-tilt, were noted. As implant-related complications, two loosened plates and one breakage of a LHS 3.5 in the diaphyseal area were observed (6%). In five cases (9%), a perforation of the DLS 3.7 occurred: One primary perforation during the surgery and four (7%) secondary perforations. Three of these secondary perforations were at least partial avascular necrosis of the humeral head. In the short post operativ observation time period we didn’t recognised any further humeral head necrosis.

No infection was observed.

## Discussion

The most important finding of the presented limited multi-centre study was the low rate of early secondary screw perforation.

However, the overall early complication rate of 22% was found to be relatively high. This was mainly related to avascular necrosis and secondary displacement of the greater tuberosity. Only in one case, a secondary screw perforation occurred without a partial avascular necrosis.

Sudkamp et al. [13] presented a multi-centre study of proximal humerus fractures after treatment with the PHILOS plate. They reported an overall complication rate of 34% and a screw perforation rate of 25%, with a primary screw perforation rate of 14% as well as a secondary perforation rate of 11%. They recorded 4% of humeral head necrosis as a reason of the perforation [13]. Egol et al. reported a perforation rate of 16%, as well as Brunner et al. [11]. Our findings are matching more with the results of Aksu et al. [14], he noted a secondary screw perforation rate of only 4.9%

Considering the selection of shorter screws, the awareness of an anatomic reduction of the tubercles and the restoring of the medial support to reduce the incidence of secondary screw perforations, two possible explanations may be given for our lower secondary perforation rate in comparison to that of Sudkamp et al. [13] or of Brunner et al. [11]:

The first reason may be the blunt tip of the DLS 3.7. The design of the screw tip of the DLS 3.7 is likely to also have a positive effect on screw anchorage and help preventing the screw from cut out. Compared to LHS 3.5 the DLS 3.7 has a round tip and fewer sharp edges at the screw tip. The sharp edges at the screw tip of the standard screw (LHS 3.5) are likely to cause higher local peak interface stresses which could promote screw loosening and cut out.

The second cause may be an *intra-* and an *inter-screw* load distribution that is due to the pin-sleeve-design of the DLS 3.7. *Intra-screw:* the force is distributed over a longer distance along the screw axis (Figure 1a). *Inter-screw:* Because of the reduction of the rigidity of the plate-screw construct, a certain damping effect occurs. The possible motion between the screw and the plate leads to a load distribution over the total number of the screws (Figure 1b). By using the locking head screws (LHS 3.5), 60% of the peak stress is applied to the two cranial plate-screws (A position of the PHILOS plate). With the DLS 3.7, the stress can be reduced to 40% in these particular screws. These findings are without any correlation to the number of used head screws although the *umbrella principle (*as many head-screws as possible) could counteract the humerus head collapse. However, the medial support certainly is an important factor [17], but in the presented data no correlation between oblique inferomedial screws (‘calcar screw’ in E position of the PHILOS plate) and screw perforation could be found.

However, there are two limitations to this study; i.e. the number of patients and the inhomogeneous patient population concerning fracture morphology, age and not evaluated of bone mineral density.

We are aware of the fact that, in this paper, we only present a short follow-up period of six months and that a screw perforation can also occur at a later point in time. But we tried to find those cases in the literature in which early screw perforations were described and compared them to our numbers.

## Conclusion

This first clinical observation study shows that the PHILOS plate in conjunction with the DLS 3.7 can be effectively used for proximal humerus fractures. The clinical results are comparable to other studies. However, the DLS 3.7 concept leads to less secondary screw perforation and therefore, it might be a successful concept in the treatment of osteoporotic proximal humerus fractures. The potential advantage of lower secondary screw perforation rate that is shown in the present study, has to be confirmed by analyzing a higher amount of patients with a longer follow-up period.

## Competing interests

The authors declare that they have no conflict of interest.

## Authors’ contributions

Conceived and designed the experiments: TF, SS, US, SD. Performed the experiments: TF, SS, US, SD. Analyzed the data: TF, SS, PM, CB, FM, TK, US, SD. Contributed reagents/materials/analysis tools:TF, SS, PM, CB, FM, TK,US, SD. Wrote the manuscript: TF, SS, PM, CB, FM, TK, US, SD. All authors read and approved the final manuscript.

## Pre-publication history

The pre-publication history for this paper can be accessed here:

http://www.biomedcentral.com/1471-2474/15/194/prepub
